# Phospholipids in Milk Fat: Composition, Biological and Technological Significance, and Analytical Strategies

**DOI:** 10.3390/ijms14022808

**Published:** 2013-01-29

**Authors:** Giovanna Contarini, Milena Povolo

**Affiliations:** Consiglio per la Ricerca e la Sperimentazione in Agricoltura–Centro di Ricerca per le Produzioni Foraggere e Lattiero-Casearie, Via A. Lombardo, 11-26900 Lodi, Italy; E-Mail: milena.povolo@entecra.it

**Keywords:** milk fat, phospholipids, sphingolipids, plasmalogens

## Abstract

Glycerophospholipids and sphingolipids are quantitatively the most important phospholipids (PLs) in milk. They are located on the milk fat globule membrane (MFGM) and in other membranous material of the skim milk phase. They include principally phosphatidylcholine, phosphatidylethanolamine, phosphatidylinositol and phosphatidylserine, while sphingomyelin is the dominant species of sphingolipids There is considerable evidence that PLs have beneficial health effects, such as regulation of the inflammatory reactions, chemopreventive and chemotherapeutic activity on some types of cancer, and inhibition of the cholesterol absorption. PLs show good emulsifying properties and can be used as a delivery system for liposoluble constituents. Due to the amphiphilic characteristics of these molecules, their extraction, separation and detection are critical points in the analytical approach. The extraction by using chloroform and methanol, followed by the determination by high pressure liquid chromatography (HPLC), coupled with evaporative light scattering (ELSD) or mass detector (MS), are the most applied procedures for the PL evaluation. More recently, nuclear magnetic resonance spectrometry (NMR) was also used, but despite it demonstrating high sensitivity, it requires more studies to obtain accurate results. This review is focused on milk fat phospholipids; their composition, biological activity, technological properties, and significance in the structure of milk fat. Different analytical methodologies are also discussed.

## 1. Introduction

Phospholipids (PLs) are basic constituents of natural membranes; their amphiphilic properties derive from the presence of both a hydrophobic tail and a hydrophilic head. This characteristic affects their role, behavior and function. They belong to the class of polar lipids and, literally, are defined as “lipids containing phosphorus”. Polar lipids are fundamental in milk for the emulsification of fat in water, because together with proteins, they are the main constituents of the milk fat globule membrane (MFGM), which encircles the lipid droplets secreted by the mammary gland cells.

Like other biological membranes, MFGM includes, together with PLs, (glyco)proteins, glycolipids (*i.e.*, cerebrosides and gangliosides), total and partial glycerides, free fatty acids and cholesterol [1].

The most representative MFGM proteins are Mucin 1, Mucin 15, CD36, Butyrophilin, Lactadherin, Xanthine Oxidoreductase, Adipophilin, and FABP, the last three unglycosylated.

Literature data on the composition of the MFGM are highly variable due to the different procedures applied for isolation and purification.

Studies applying laser confocal scanning microscopy provide interesting information about the structure and the lateral organization of MFGM.

MFGM is trilaminar, with a first surface-active layer, mainly consisting of proteins, surrounding the intracellular neutral lipids. This inner part is covered by a bilayer membrane deriving from the secretory cell apical plasma membrane [2]. PLs are mainly located in this outer leaflet and are organized as a liquid-disordered phase coexisting with a liquid-ordered phase (also called a lipid raft), the latter rich in sphingomyelin and cholesterol [3,4].

The interest in these molecules is high due to both the possible positive effects on human health [5–7] and their technological properties in the food industry as emulsifiers or emulsion stabilizers [8]. Milk and some by-products of dairy production are an interesting natural source of PLs.

In this paper, the state of the art on milk fat PLs is reviewed with respect to their structure, composition, biological activities, technological properties and analytical techniques.

## 2. Nature and Characteristics of Phospholipids in Dairy Products

**Figure 1:**
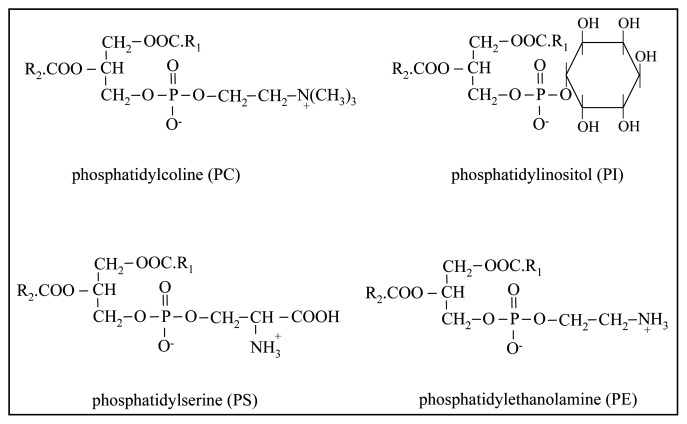
Structures of the principal glycerophospholipids in milk fat.

Glycerophospholipids and sphingolipids are the two main groups belonging to the class of PLs. Glycerophospholipids are formed by glycerol, phosphoric acid, fatty acids and a hydroxy compound (e.g., choline, ethanolamine, serine, inositol). Figure 1 shows the structure of the most important PLs of milk fat: phosphatidylcholine (PC), phosphatidylethanolamine (PE), phosphatidylinositol (PI) and phosphatidylserine (PS). The two fatty acids, mainly represented by unsaturated FAs, are esterified at the *sn-1* and *sn-2* positions of the glycerol backbone.

**Figure 2:**
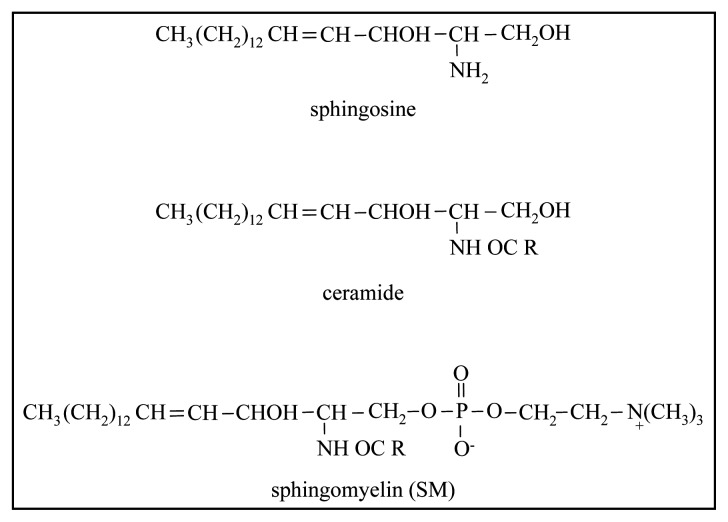
Structures of the sphingomyelin and its derivatives.

Sphingolipids are compounds containing a long chain base, the so called sphingoid base (e.g., sphingosine or phytosphingosine), fatty acids and sugars or phosphoric acid or alcohols [9]. Sphingosine is the principal sphingoid base in mammalian sphingolipids, forming a ceramide when its amino group is linked (amide bond), generally, with a saturated fatty acid. Sphingomyelin (SM) is the dominant species and it is composed of a phosphorylcholine head group linked to the ceramide (Figure 2).

**Figure 3:**
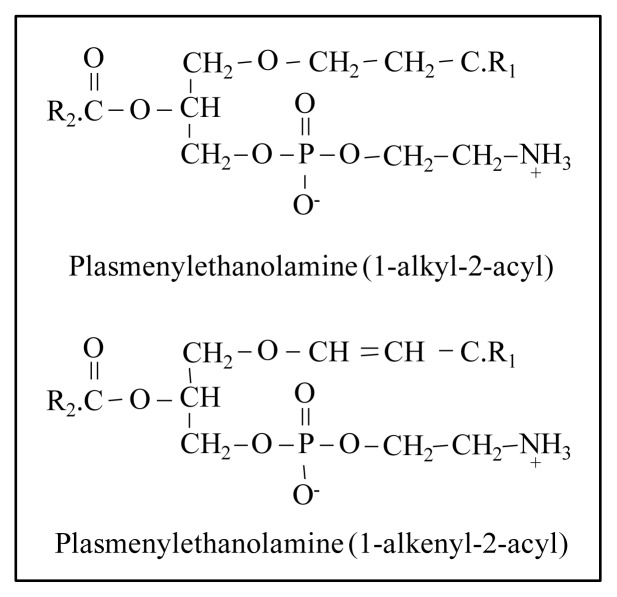
Structures of plasmalogens subclasses of phosphatidylethanolamine (PE).

Among minor PLs in milk, some authors detected lysophosphatidylethanolammine (LPE), lysophosphatidylcholine (LPC) and plasmalogens (Figure 3) [10–13]. LPE and LPC derive from partial hydrolysis of PC and PE. Their origin is still uncertain though, because they could be formed as a consequence of a hydrolysis process taking place during the handling of milk (pumping and keeping it at a refrigerating temperature for a few hours). Plasmalogens are glycerophospholipids with ether-linked alkyl chain at the *sn-*1 position instead of the ester linked fatty acid. Some of the ether-linked phospholipids also display a *cis* double bond on the alkyl chain, forming a “vinyl-ether linkage” [14]. These molecules are widely distributed in many animal tissues. In particular, the interest in these compounds is growing as they are abundant in brain and human heart tissue and seem to be linked with some pathologies and with human genetic disorders [14]. In animal tissues the principal plasmalogens are usually in the PE class, less in the PC class and little or absent in the other groups. As for the role of these compounds, they are constituents of the cell membranes but the absence of a carbonyl oxygen in the *sn*-1 position makes their structure much more lipophilic and leads to changes to the arrangement of lipids within membranes. As a consequence, plasmalogen-containing cell membranes are less fluid than those deficient in plasmalogens [15].

Glycerophospholipids and sphingolipids are quantitatively the most important PLs in milk. They represent about 0.5%–1% of milk fat and about 60%–70% of the PL in milk are in the MFGM, placed mainly in the external bilayer membrane [3]. The origin of PLs, as most of the MFGM, is the apical plasma membrane of the mammary gland secretory cell [6]. It is hypothesized that most PLs containing choline (PC) and SM are located on the outside of the membrane, whereas PE, PS and PI are mainly placed on the inner surface of the membrane [16].

In the PLs of milk fat the short and medium chain length FAs are nearly absent and, in particular, PE is highly unsaturated, whereas PC contains more saturated FA in comparison with the other PLs [6]. Fong *et al.* [17] detected only a small amount of very long chain FAs (carbon atoms >20) esterified in the glycerophospholipids PE, PC, PI and PS. Completely different is the FA composition of dairy SM: the main FAs are C16:0, C18:0, C18:1n9, C22:0, C23:0 and C24:0, thus it is a highly saturated PL [12,17,18]. The particular FA composition of SM provides this molecule with the ability to form in the cellular membranes, together with cholesterol, rigid domains, called “lipid rafts”, involved in different important cellular processes. This behavior is possible because the structure has a higher melting point and degree of packing in comparison with the domains where the glycerophospholipids are present [6].

An overview of the total polar lipid content of milk, together with the detailed PL class composition, obtained by several authors, is reported in Table 1. In order to render the different papers more uniform, the total PL amount was calculated, where necessary and possible, with respect to the fat content by using the total lipid value reported in each paper. Moreover the percentage of the five main PLs was recalculated excluding the other phosphorous-based molecules, if detected. The total amount of polar lipid content ranges from 0.25 to 0.96 g/100 g of fat. This variability can be explained by differences in the extraction and analysis methods as well as by other factors, such as the diet of the animal [19] and the period of lactation [20]. The most abundant PLs in milk fat, expressed as percentage of total PLs, are PE (26.4%–72.3% of total PL), PC (8.0%–45.5% of total PL) and SM (4.1%–29.2% of total PL) followed by PI (1.4%–14.1% of total PL) and PS (2.0%–16.1% of total PL). Graves *et al*. [18] observed a relation with the breed regarding SM, the most interesting PL under the biological point of view. A higher content of SM was detected in milk fat from Holstein cows than Jersey, suggesting that the greater fat globule size in Jersey milk is responsible for this difference. Graves *et al.* [18] concluded that its content increased with the stage of lactation and during summer.

Less numerous are the studies aiming to determine the PL composition of milk of different mammalian species (Table 2). Rodriguez-Alcalà *et al.* [28] detected statistically significant differences among species and in particular between cow and ewe’s milk. Both Benoit *et al.* [32] and Garcia *et al.* [13] analyzed human milk and found a lower percentage of PE and a higher percentage of SM with respect to the milk of the other species. Moreover Garcia *et al.* [13] observed that the human milk was richer in PLs classes and, in particular, in phosphatidylethanolamine plasmalogens, with respect to the other species. The composition of PLs of donkey milk, reported by Donato *et al.* [30], appeared extremely different from that of milk of all the other species, particularly for the high values of PE and the low values of SM. However it is worth noting that even the values of cow milk (Table 1) were very far from those obtained by the other authors, suggesting possible problems related to the analytical method applied.

Not only are the characteristics of the raw material, but also the technological process applied, responsible for the PL content of dairy products. Being PLs mainly present in the MFGM, any treatment that produces a disruption of the membrane and/or a fractionation or separation of fat globules (e.g., centrifugation) and/or of polar and neutral components of fat, can affect the distribution and composition of PLs in the final matrix [12,24]. Thus, cream has a polar lipid content (expressed on the total lipids) lower than skimmed milk, just as butter and cheese have a lower polar lipid content with respect to buttermilk and whey (Table 3).

## 3. Biological Activity and Health Effects

The beneficial effects of dietary PLs concerning heart diseases, inflammation and cancer would seem to have been known since the early 1900s. Sphingolipids are abundant in the apical membrane in the absorptive epithelium in the gut [36], and their digestion products (ceramides and sphingosine) are considered as the most bioactive compounds, having important effects on cell regulation. These compounds are critical for the maintenance of membrane structure, modulating the behavior of growth factor receptors and serving as binding sites for some microorganisms, microbial toxins and viruses [37]. Ceramide is a major lipid messenger that inhibits cell proliferation and induces apoptosis, whereas sphingosine-1-phosphate is a second messenger inside the cell and there is evidence that indicates the important role of the latter molecule in regulation of cell growth, angiogenesis, immune function and lymphocyte traffic [38].

It has been estimated that the average human dietary intake of PLs is 2–8 g/day, introduced with different types of food, such as eggs, cereal grains, oilseeds, fish, beef and cow’s milk [39]. Dairy products are most likely to be the major source of sphingolipids. In the US diet, 116 g (calculated as SM), corresponding to 0.01%–0.02% *w*/*w* of the diet and 0.3–0.4 g/day, is the yearly intake per capita [37].

The oral application of dietary PLs with a specific FA composition seems able to modify the FA composition of the membrane of a certain cell type, modulating thus the cellular functions, as well as the activity of membrane bound enzymes [7].

Researches on the positive effects of PLs on membranes have been carried out *in vitro*, since it is very difficult, especially in humans, to perform these studies *in vivo*. The following is a review of the main diseases where the beneficial effects of milk PLs have been seen.

### 3.1. Cardiovascular Diseases

Recent studies showed that the increased consumption of milk and dairy products is associated with a reduced incidence of obesity, insulin resistance, dyslipidemia and type 2 diabetes, that are cardiovascular risk factors [40,41]. Wat *et al.* [42] investigated the effect of milk-derived PLs on the lipid metabolism of plasma and liver in mice, and observed that the supplementation of the high-fat diet with a phospholipid-rich dairy milk extract caused a significant decrease of the liver weight, total liver lipid, liver triglycerides and total cholesterol and serum lipids. Similar results on mice were obtained by Watanabe *et al.* [43]. Studies carried out by Ohlsson *et al.* [44,45] on humans, consisting of the supplementation of a sphingolipid-enriched dairy formulation, partially supported the findings that these molecules may affect cholesterol concentrations in TG-rich lipoproteins, but they did not find any effect on the level of plasma lipids or lipoproteins. Keller *et al.* [46] concluded that milk PL supplementations influenced the plasma cholesterol amount, but did not change the LDL/HDL ratio.

Nevertheless, PL human metabolism, through the group of phospholipases A_2_ (PLA_2_s) enzymes, can produce molecules that are associated with cardiovascular diseases onset. These enzymes are esterases that catalyze the hydrolysis of glycerophospholipids at the *sn*-2 position, producing non-esterified fatty acids and lysophospholipids. PLA_2_s can be cytosolic or extracellular (associated with lipoproteins: Lp-PLA_2_; or secreted: sPLA_2_) enzymes [47]. What their role is on human health can be very different, depending on the enzyme. One of the sPLA_2_ enzymes has a function in the digestion of dietary PLs, another in host defense against bacterial infections, but there is some research that suggests the involvement of other enzymes in promoting atherosclerosis and cancer [47–49].

### 3.2. Inflammation and Gastrointestinal Infections

Inflammation is a response to harmful stimulus, in order to remove it and start a healing process. The mucosal surface of the digestive tract represents a barrier between a wide spectrum of potentially harmful factors [50], and polar lipids were effective in protecting cellular membranes. In a recent research by Veereman-Wauters *et al.* [51], the consumption of a MFGM-enriched milk by young children has seen to have a protective effect against gastrointestinal infections, producing a significant decrease in the number of short febrile episodes.

Studies have suggested an activity of PLs in regulating the inflammatory reaction. Dial *et al.* [52] attributed the antiulcer action of milk to the protective role of dipalmitoyl lecithin. A further study, conducted on human subjects, demonstrated that the acetylsalicylic acid (ASA) induced injury to the gastric mucosa, was markedly reduced or completely abolished if ASA was chemically associated with the PC [53].

In addition, several researches of Sprong *et al.* [54–56] highlighted the positive effect, *in vitro*, of both C10:0, C12:0, unsaturated C18 fatty acids and sphingolipids against gastrointestinal infections. In particular, 100 mmol L^−1^ of lysophingomyelin was highly bactericidal against *Campylobacter jejuni*, *Listeria monocitogenes* and *Clostridium perfringens*, and moderately lowered viable counts of *E. coli* and *Salmonella enteritidis*. In addition, 100 mmol L^−1^ of sphingosine decreased viable counts of all pathogens tested.

PC was even found to be active in reducing the development of arthritis. Hartman *et al.* [57] demonstrated that exogenous PC ameliorates leukocyte-mediated signs of acute arthritis, and the outcome is comparable to that of non-steroidal anti-inflammatory drug treatment in the short term. The analgesic properties of PC were associated with reduced joint swelling, and decreased leukocyte adhesion and infiltration at the level of the synovial microcirculation. The study of Eros *et al.* [58] provided evidence that an increased dietary PC uptake prior to collagen-induced arthritis was associated with significantly enhanced anti-inflammatory protection. The same authors then suggested that the use of phosphatidylcholine-enriched food as a pretreatment, but not as a therapy, could exert beneficial effects on the morphological, functional and microcirculatory characteristics of chronic arthritis.

### 3.3. Stress Conditions

One central stress-regulating system is the hypothalamic-pituitary-adrenal axis (HPAA), which allows adaptation to stressful challenges by releasing the glucocorticoid cortisol via corticotrophin-releasing hormone and adrenocorticotropic hormone (ACTH). Stress-buffering effects of PLs have been related to dampened plasma cortisol responses to short-term exercise and mental stress [59].

Milk-based PLs have shown positive effects on individuals under chronic stress, improving the ability of the organism to adapt to the condition, increasing the cortisol availability and attenuating stress-induced memory impairments. A dose of 1% PL, administered as concentrated milk PL, protected people who were continually exposed to chronic stress with respect to both physical and mental health [60].

Hellhammer *et al.* [61] investigated whether the daily intake of PL concentrate rich in PS and SM had similar beneficial effects on working memory and the acute stress response. A better response with a tendency for shorter reaction times in the working memory was observed in PL-treated subjects, compared to placebo-exposed individuals. The two treatment groups did not significantly differ in their endocrine stress response. However, PL-treated subjects with a higher stress load showed a blunted psychological stress response.

### 3.4. Cancer

Ceramides and sphingosine, the digestion products of sphingolipids, affect cell growth, differentiation and apoptosis, suggesting that their release may have an effect on the behavior of normal or transformed cells, especially of the intestine [38].

Cancer cell membranes acquire particular properties, which vary from those found in the differentiated progenitor cells. The membrane of breast and prostate cancer cells was shown to have a higher concentration of “lipid rafts” than their normal counterpart cells, which was associated with higher apoptotic sensitivity. As above-mentioned, SM, due to its content of long chain saturated fatty acids, highly contributes to form, together with cholesterol, the rigid domains, called “lipid rafts”. Consequently, the regulation of the composition and density of lipid rafts could potentially alter cancer cell viability and metastatic behavior [7].

Non-pharmacological amounts of SM in the diet showed chemopreventive and chemotherapeutic effects on chemically induced colon cancer in mice [62].

Studies on other cell types indicate that sphingolipids may have a protective activity even against damage from γ-irradiation and chemical agents [37].

Finally, the research of Russell *et al.* [63] suggests that milk PLs, and in particular SM, act upon skin cells protecting them against the effect of ultraviolet radiation.

The impact of lipid components of the MFGM on human health, particularly in relation to colorectal cancer has recently been reviewed by Kuchta *et al.* [50].

### 3.5. Cholesterol Absorption

PLs also seem to have an effect on cholesterol intestinal uptake [64]. Kamili *et al.* [65] in a study with mice fed a high-fat diet observed that milk PLs reduce the hepatic accumulation of intestinal cholesterol and increase the fecal cholesterol excretion. In particular, the associations between SM and cholesterol may be responsible for this behavior, since the two molecules in the cellular membranes are localized in the same domain [66]. SM affects different aspects of cholesterol transport and metabolism suggesting that it may influence atherosclerosis [37]. Noh *et al.* [67] observed that milk SM is a more potent inhibitor of the intestinal absorption of cholesterol than egg SM and this behavior can be explained by the higher degree of saturation and longer chain length of the fatty acyl groups.

### 3.6. Nervous System Myelination and Neurological Development

SM and sphingolipid metabolites are fundamental components in the central nervous system of myelin sheath that surrounds the axons of some neurons. The importance of the myelination process in the development of the human brain is confirmed by the fact that it begins at 12–14 weeks of gestation and continues up to the second postnatal year [68]. A research of Oshida *et al.* [69] on developing rats demonstrated that dietary SM can contribute to the myelination of the central nervous system; furthermore the study of Tanaka *et al.* [70], carried out on premature infants, showed that the administration of SM-fortified milk to the babies had a positive association with the neurobehavioral development.

PLs are carriers of essential polyunsaturated FAs, very important molecules for the fluidity of the membrane. This function is crucial during aging because in this period the lipid composition of brain cells changes and the content of polyunsaturated n3-FAs decreases. PC has also a role as antioxidative agent in the treatment of alcohol induced brain changes and choline affects brain development and lifelong memory characteristics [7]. Finally, there are studies supporting the hypothesis that dietary PLs could contribute in the therapeutic approach to Alzheimer’s disease, even if this is still a controversial issue. Neurodegenerative diseases, such as Alzheimer’s and Parkinson’s, are caused by endoplasmic reticulum stress [71]. Nagai [72] demonstrated the protective function of milk PLs on endoplasmic reticulum stress induced neuronal cell death, and concluded that consumption of milk PLs or milk products may reduce the risk of some neurodegenerative diseases.

## 4. Technological Properties

The emulsifying properties of PLs are due to the simultaneous presence in the molecule of a water-loving hydrophilic head and an oil-loving hydrophobic tail. As above reported, milk PLs are mainly located in MFGM in association with protein and other polar lipids. Because of its original function in emulsifying the fat globules in whole milk, MFGM material isolated from buttermilk or cream is considered to be an efficient natural surface-active material, highly effective in lowering the interfacial tension [1].

The studies aiming to exploit the technological properties of milk PLs are thus based on the purification of MFGM from dairy by-products showing the highest concentration of MFGM fragments, particularly buttermilk and butter serum.

Buttermilk is the aqueous phase released during the churning of cream in the manufacturing of butter. This definition includes a wide range of milk fat by-products, according to the raw material used, the pre-treatment conditions and the butter making process [73]. The most common type of buttermilk is sweet buttermilk deriving from churning sweet cream into butter. However, other types of buttermilk can be produced from milk fat, such as sour buttermilk, produced from churning cultured cream, in the manufacture of European-style butter; or whey buttermilk from churning whey cream, obtained by centrifugation of whey deriving from cheese making [74].

Butter serum is a by-product of anhydrous milk fat production. The anhydrous milk fat is produced by melting and centrifuging butter (or a 75% cream can be homogenized, forcing phase inversion), resulting in pure butterfat and butter serum. The composition of butter serum is comparable to that of buttermilk, except for the fat content (2.6%–3.7%), which is much higher than buttermilk (0.5%–1.5%). The phospho- and sphingolipid contents of butter serum (0.9–1.2) are much higher than those of buttermilk (0.1–0.2) [75].

Whole buttermilk contains, together with MFGM fragments, skim milk-derived proteins (casein and whey proteins), lactose and minerals. Therefore, purification processes, mainly based on membrane filtration, are needed to separate the MFGM material. Corredig *et al.* [76] carried out several ultrafiltration, microfiltration and diafiltration experiments and evaluated the ratio of concentration of MFGM constituents together with the degree of purification. The authors concluded that microfiltration, through a 0.1 μm filter, of commercial buttermilk, in which casein micelles were disrupted by the addition of sodium citrate, was the most effective procedure to obtain an isolate containing a high ratio of MFGM material.

The emulsifying properties of the reconstituted microfiltered buttermilk (MF-BM: 9.3% PLs on dry matter), in comparison with those of buttermilk powder (BMP: 3.3% PLs on dry matter), skim milk powder (SM: 0.2% PLs on dry matter) and sodium caseinate (SC: PLs not determined), were evaluated by Phan *et al.* [77]. Mixtures prepared with of 35% soybean (Oil/Water), were homogenized at different pressures. The emulsions prepared with MF-BM and SC showed a narrower particle size distribution compared with that of emulsions prepared with BMP and SMP, and those containing MF-BM were the most stable, among the four types of emulsions.

In addition, MF-BM emulsions had very low viscosity and Newtonian-like flow characteristics. The authors confirmed that the highest concentration of PLs in MF-BM was responsible for its higher emulsifying/stabilizing activity, in comparison with the other materials.

MFGM fragments have also gained considerable attention for their ability to improve the heat stability of recombined evaporated milk emulsions [78]. Two powdered dairy by-products, sweet buttermilk (SBP) and the cream residue after the production of butter oil (CRP), were added, in different concentrations, to 16.5% (*w*/*w*) skim milk powder, 6.5% (*w*/*w*) sunflower oil and 77% of an aqueous solution with 0.02% NaN3, to obtain recombined evaporated milk emulsion samples.

The recombined samples were then heated at 121 °C for 15 min. Viscosity and particle size distribution measurements revealed that MFGM-enriched and hence PL enriched dairy by-products were able to avoid the whey protein aggregation as well as the whey protein-casein interaction. This usually occurs during the sterilization of the recombined evaporated milk. These results lead to the hypothesis that PL-enriched dairy by-products can replace exogenous ingredients, such as soybean lecithin or alternative surfactants, in recombined milk emulsions.

MFGM components can also play a role in the transport and delivery of liposoluble ingredients in the gastrointestinal tract. Bezelgues *et al.* [79] observed, by an in vitro digestion study with tocopherol and lycopene, that MFGM-purified fraction had a higher ability in the transfer of liposoluble molecules into bile salts micelles than other conventional emulsifying milk proteins.

Milk PLs have been successfully tested for the preparation of liposomes, PL bilayer vesicles used both for the encapsulation and controlled release of bioactive compounds and for enhancing their stability and bioavailability [80,81]. Gülseren *et al.* [80] applied milk PLs for the encapsulation of polyphenols with the aim to optimize the delivery of these potential bioactive molecules. Farhang *et al.* [81] used these molecules for the encapsulation of ascorbic acid, a very labile compound that can be used as both a vitamin supplement and an antioxidant.

## 5. Analytical Strategies for PL Determination

A significant effort has been made in the past decades to develop quali-quantitative methods for determining phospholipid composition of milk and dairy products.

### 5.1. Extraction of Fat from Milk and Dairy Products

Compared to other food, milk is a very complex matrix with a high amount of water and similar content of fat, protein and lactose. PLs interact with both lipids and proteins, due to their amphiphilic properties, and therefore particular care should be taken during fat extraction to be sure to recover the whole lipidic fraction.

The Rose Gottlieb principle, which includes a digestion with ammonia and an extraction with a mixture of diethyl and petroleum ether, and the Schmid-Bondzynski-Ratzlaff principle, which applies the same solvent mixture, but a digestion with hydrochloric acid, are the ISO official methods adopted for the quantitative extraction of fat from milk [82] and cheese [83]. This solvent mixture was not really reliable for the PL extraction [84], particularly regarding PS and PI [22]. The most applied procedure to obtain a lipidic fraction suitable for the PL determination, is based on the extraction with a solvent mixture including chloroform and methanol.

The use of this mixture arises from the studies aiming to extract PLs and SPLs from biological materials, particularly animal tissues [85,86]. Both procedures start with the addition of a chloroform/methanol mixture. The initial solvent system is binary; during the extraction process, it becomes a ternary system consisting of chloroform, methanol, and water in various proportions, depending on the moisture content of the sample. The Folch procedure [85] uses a solvent-to-sample ratio of 2:1 (*v*/*w*) with a mixture of chloroform and methanol (2:1 *v*/*v*) in a two-step extraction. The extract is then diluted with a salt solution until the phases separate and the lower phase containing lipids is collected. Bligh and Dyer [86] use a 1:1 (*v*/*v*) chloroform–methanol mixture for the first step extraction, and the ratio is then adjusted to 2:1 (*v*/*v*) in the alternate steps of extraction and washing. Moreover, in this procedure, the amount of water naturally present in the sample is specifically taken into account, and the amount of sample should be adjusted to obtain, in the first extraction step, a ternary system chloroform/methanol/water with a ratio of 1:1:0.9 (*v*/*v*/*v*).

Both procedures use large amounts of sample and solvents; therefore, some authors provided modifications aiming to reduce the amount of solvents and sample [87], to improve the extraction of lysophosphatides and plasmalogens [88] and to adapt the system to the dairy products [21,29,89]. Recently, the accelerated solvent technique has been applied to cheese samples to enhance the capabilities of conventional solvent extraction [90]. The extraction was carried out with ethyl acetate/cyclohexane (54:46, *v*/*v*) and methanol/ethyl acetate (1:1, *v*/*v*) and the recovery, calculated by spiking the samples with PC standards, ranged between 87.8% and 105%.

### 5.2. PL Separation from Lipid Matrix

Due to the low concentration of PLs in milk and dairy products (0.3–1 g/100 g of fat), except for dried butter serum and buttermilk, a second extraction step is often applied to separate PLs from the other fat constituents, mainly triglycerides.

Lipid extracts are usually fractionated by column chromatography on a preparative scale before subjecting them to detailed analysis. The solid–liquid chromatography, in which the elution of the desired lipid class is achieved by varying the polarity and strength of the mobile phase, is the most applied technique for PL separation. The traditional glass columns of diameter of few cm and heights of not more than 40 cm, are nowadays substituted by prepacked commercial columns, named SPE (solid phase extraction), which require less time, solvent, and packing material.

Table 4 reports the different types of SPE sorbents applied for the PLs purification. Both normal phase (silica and NH_2_) and reversed phase (C8 and C18) chromatography were used by the different authors. When different sorbents were compared [12,22,91], normal phase SPE with silica sorbent provided the most accurate results, except for Caboni *et al.* [91] who obtained the highest yield by using the C8 SPE.

Normal phase SPE procedure typically involves a polar analyte, a mid-to non-polar sample solvent and a polar stationary phase. Retention of PLs, under normal phase conditions, is primarily due to interactions between polar functional groups of PL and polar groups on the sorbent surface. Non polar compounds are eluted in the early step of the procedure, whereas PLs interact with the polar groups on the sorbent surface. The disruption of this binding mechanism, *i.e.*, the elution of PLs, is obtained by applying a solvent that is more polar than that in which sample is dissolved. Exactly the opposite behavior occurs when reverse phase SPE is applied.

Thin layer chromatography (TLC) is one of the other chromatographic methods frequently used for the fractionation of complex lipid mixtures. It is based on the difference in the affinity of a component toward a stationary, generally silica gel, and a mobile phase. In comparison with SPE and HPLC, TLC is more time consuming and less quantitatively precise. Nowadays, this technique is applied principally for testing the effectiveness of other methods of separation, e.g., SPE [12], for qualitative [34] and for preparative purposes [18,26,27,32].

It can be used to separate both PLs from the whole lipidic matrix, and the single PL species.

The separation of polar lipids from neutral lipids was obtained applying a mobile phase composed of hexane/ether/acetic acid [32] or acetone [18]; the detailed separation of the single PLs was performed by using: chloroform/methanol/methylamine [26,32], or chloroform/methanol/water [12,18].

A two-dimension TLC, using chloroform/methanol/ammonia/water for the first dimension and chloroform/methanol/acetic acid/water for the second one, was adopted by Sanchez-Juanes *et al.* [27] to obtain pure PL constituents.

### 5.3. Quantification and Identification of Single PLs

#### 5.3.1. HPLC Coupled with ELSD or MS

High performance liquid chromatography (HPLC) with evaporative light scattering detection (ELSD) is the most widely chromatographic system used for PL analysis in the dairy sector (Table 5).

ELSD is a mass flow sensitive detection method suitable for non-volatile sample components, without significant chromophore groups, in a volatile eluent. The eluent stream passes through a nebulizer into an evaporation chamber, where the solvent is evaporated to leave a mist of tiny sample particles. These scatter a light beam, and the extent of the light scattering is proportional to the amount of sample present.

To maximize the response and the linear range, particular care should be taken to adjust the flow rate of the nebulizer gas and the temperature of the evaporator chamber. Moreover, good quantitative results could be obtained if the calibration conditions were rigidly set to be the same as in the analysis of real samples.

HPLC coupled with ELSD was applied both on the PL purified fraction [22,30,91] and directly on the whole fat extracted from milk and dairy products [25,28,29].

The HPLC separation of PLs was always performed in normal phase by using a silica column, except Donato *et al.* [30] who used a hydrophilic interaction liquid chromatography (HILIC). It is a particular version of normal phase liquid chromatography that uses a hydrophilic stationary phase and can be performed with partially aqueous mobile phases. With respect to the traditional normal-phase chromatography, HILIC has the double advantage of using water-miscible solvents, which are compatible with electrospray ionisation detection (ESI), making on-line hyphenation to mass spectrometry (MS) detection straightforward.

An alternative HPLC detector, charge aerosol detector (CAD), based upon aerosol charging, has been recently applied by Kielbowicz *et al.* [31] to milk PL analysis. Its principle of operation is based on charging aerosol particles by corona discharge and subsequent measurement of the charged particles using an electrometer. CAD adopts the same mobile phase nebulization principle as ELSD but uses a charge transfer for solute detection that makes this detector more sensitive and precise than ELSD [92].

Several mobile phases were adopted, all in gradient mode. Except for Rombaut *et al.* [25], the other analytical procedures included more than one solvent mixture, and Rodríguez-Alcalá [28] even a quaternary gradient.

The identification by mass spectrometry detector (ESI-MS/MS) was also adopted by Gallier *et al.* [12] to assess the accuracy of different extraction/purification methods of PLs and to measure the effect that processing had on PL composition. In that paper the extracts from the three SPE conditions tested (Table 4), were directly introduced by continuous infusion into the ESI source on a triple-quadrupole MS/MS. This technique allowed the detection, in dairy products, of a large number of PL molecules (LPC, PC, SM, ePC, LPE, PE, PE-cer, ePE, PI, PS, PA).

#### 5.3.2. NMR Technique

Phosphorus occurs predominantly as the isotope ^31^P, which has a nuclear spin value of 1/2 and is therefore visible in nuclear magnetic resonance (NMR) spectrometry. The ^31^P NMR method is widely used, in such diverse areas as the characterization of organic and inorganic molecular structures and the analysis of biological fluids, including milk and derivatives.

The large range of chemical shifts (about 700 ppm) reported for the ^31^P nucleus, its 100% natural abundance, and its high sensitivity, which is only about 15 times less than that of the proton nucleus, makes ^31^P NMR a reliable analytical tool to determine very low concentrations of molecules containing phosphorous [93]. Nevertheless, NMR is still an expensive instrument and requires very experienced analysts.

Several authors [11,13,23,34] applied ^31^P NMR to dairy products, mainly milk, to evaluate the PL constituents (Table 6).

Murgia *et al.* [11] identified and quantified PLs in both ewe and cow milk cream, obtained from milk centrifugation. The polar lipid fraction was then separated by applying the Folch extraction method. They compared the traditional NMR solvent mixture including chloroform/methanol/water-EDTA with a monophasic mixture composed of dimethylformamide/triethylamine/guanidinium hydrochloride. The monophasic mixture seemed to solve some of the partition problems related to the traditional biphasic system, and slightly improved the PL resolution, but caused the formation of some adducts leading to an underestimation of PE. Together with the PLs reported in Table 6, the analytical procedure applied by Murgia *et al.* [11] provided the qualitative detection of LPC, MMPE, and LPE.

Andreotti *et al.* [23] applied ^31^P NMR to recognize not only the PL composition of buffalo milk in comparison with cow milk, but also the composition of the other phosphorilated compounds. They applied the same monophasic mixture as Murgia *et al.* [11] and, together with the PL composition of the lipidic fraction extracted by the Folch method [85], analyzed both the whole milk and the defatted milk, ten fold concentrated by ultrafiltration. No significant differences were observed in the PL composition of fat fraction, between the two types of milk. Total inorganic P, glycerophosphorylethanolamine and glycerophosphorylcoline were detected by the ^31^P NMR analysis of whole milk; in addition, *N*-acetylglucosamine-1-phosphate, galactose-1-phosphate, phosphorylcholine, phosphorylethanolamine, glycerol-1-phosphate, and glucose-6-phosphate, were identified in ultrafiltered milk.

A comparison between the quantitative two-dimensional thin-layer-chromatography (2D-TLC) and ^31^P NMR was performed by MacKenzie *et al.* [34], on different types of dairy products: cream, fresh beta serum, and buttermilk protein concentrate powders. TLC method appeared more sensitive and detected a number of compounds not seen by NMR. Nevertheless, when the pH of the detergent (sodium cholate/EDTA) was raised 7.1 to 9.5, major phospholipids PC, PE, SM, DHSM, PI and PS were correctly measured by NMR. Comparable NMR results were also obtained by analyzing both the lipid fraction and the whole sample of some concentrated dairy products, providing that they have a high PL content and a good solubility in the NMR detergent.

Results on the PL composition of cow, camel, mare and human milk, analyzed by ^31^P NMR, were recently reported by Garcia *et al.* [13]. Among the parameters checked to optimize the NMR analysis, the temperature significantly affected the spectral resolution. The temperature appeared specifically correlated with the milk species, even though the authors did not provide any explanation for this behavior. The optimized method proposed by Garcia *et al.* [13], was suitable for determining the largest number of bioactive phospholipids, including plasmalogens.

Despite its high sensitivity and the simple pulse sequences that are used, the ^31^P NMR analytical technique needs more detailed studies to be accurately applied for the determination of PL composition of dairy products. Sample preparation, solvent mixtures, pH and temperature were the most critical parameters investigated by the different authors and for which new data are certainly advisable.

### 5.4. Determination of the FAs Bonded to PL Molecules

The FA composition of the PLs is carried out by two approaches: by GC and GC/MS of methyl esters of PLs previously purified by different techniques [26,27,32,90], and directly by LC/MS [12,17,30]. As for the application of LC/MS technique, the main results were related only to the qualitative occurrence of FAs in the PL molecules. Gallier *et al.* [12] discriminated the FAs only on the basis of the degree of unsaturation, whereas Donato *et al.* [30] and Fong *et al*. [17] provided more detailed indications about the type of fatty acid esterified in the PL molecules.

As far as the derivatization process is concerned, different methods can be applied [94]. Base-catalyzed methods, e.g., with sodium or potassium methoxide in methanol, can be adopted, but they do not esterify the amide-bound fatty acids as in sphingolipids. One of the most used approaches is the acid-catalyzed transmethylation by boron trifluoride in methanol. This method has the advantage that it esterifies even the free fatty acids, if present, but it has the disadvantage of being able to produce many side effects and for this reason it should be avoided. Methanolic hydrogen chloride and methanolic-sulfuric acid are considered the best general purpose esterifying agents [94].

## 6. Conclusions

The studies carried out on milk PLs, glycerophospholipids and sphingolipids, clearly showed that these molecules play a key role in both the nutritional and technological field.

The low amount of PLs in milk together with their amphipilic chemical structure, are responsible for the numerous efforts made by different researchers to improve the methodologies for the analytical determination of these compounds. However, milk fat extraction, PLs separation from lipidic matrix, detailed identification and precise quantification, still remain a not completely solved problem. It is not surprising that a standardized method for milk PLs determination is not yet available and it is certainly one of the main causes of both the variability of the results and the difficulties of comparison among the different researches. A reliable analytical procedure would be essential for supporting the evidence of the beneficial effects of milk PLs on the human health.

Finally, it would be advisable to enhance the development and the application of some dairy by-products, e.g., buttermilk, which, due to the high content of PLs and reduced amount of triglycerides, have potential health properties.

## Figures and Tables

**Table 1:** Polar lipid content (g/100 g of fat) and phospholipids composition (percentage of total phospholipids (PLs)) of liquid milk.

Reference	Polar lipids	PE	PI	PS	PC	SM	Note
[21]	0.69	38.6	-	-	32.2	29.2	1
[22]	0.36	32.3	9.3	10.5	27.3	20.5	
[23]		26.9	13.7	4.1	27.5	27.7	
[24]	0.96	33.2	5.2	9.3	27.4	25.1	
[25]	0.7	46.4	5.3	7.4	21.1	19.8	
[17]		32.6	7.6	5.3	33.2	21.3	
[26]		36.4	7.6	6.5	32.1	17.3	
[19]	0.25–0.30	26.8	13.6	16.1	22	21.6	
[27]	0.48	28.5	14.1	-	32.7	23	1,2
[28]	0.36	38.5	6.5	7.7	25.9	21.4	
[12]		26.4	3.4	2	42.8	25.5	
[29]	0.69	36.9	6.1	6.3	27	23.7	
[30]		72.3	1.4	11.5	8	7.9	
[13]		33.8	3.9	10.6	30.5	21.2	
[31]	0.65–0.89	34.2	7.7	8.6	45.5	4.1	1

1 the total amount of polar lipids was calculated considering an average fat content of cow milk of 3.5%.

2 The percentage of PI includes also PS.

**Table 2:** Content of main PLs in milk fat of different mammalian species. Values are expressed as percentage of total PLs, applying the same calculation reported for Table 1.

Reference	Species	PE	PI	PS	PC	SM	Note
[23]	buffalo	24,5	19,7	6,6	24,3	24,9	
[28]	goat	31.7	6.3	8.3	28.5	25.2	
	ewe	34.4	4.4	5.2	28.6	27.4	
[32]	human	21.3	16.4		19	43.3	1
[30]	donkey	60.2	2.4	11.2	17.3	8.8	
[13]	mare	24.3	8.5	10.6	27.8	28.9	
	human	21.7	4.5	9.6	29	35.2	
	camel	34.3	4.9	10.5	22.1	28.1	

1 The percentage of PI includes also PS.

**Table 3:** Polar lipid content (g/100 g of fat) and phospholipids composition (percentage of total PLs) in milk fat of dairy products and by-products of butter-making process.

Reference	Matrix	Polar lipids	PE	PI	PS	PC	SM
[22]	cream	0.86	42.7	6.8	7.2	14.6	28.6
	butter	0.2	31	11.9	15.3	24.7	17.1
	buttermilk	4.49	33.5	2.4	10.3	35.5	18.3
[33]	cow cream	0.17					
	cow buttermilk	0.17	38.7	9.3	9.1	23.9	18.9
	cow butter serum	0.88	27.2	10.8	7.2	29.8	24.9
	goat cream	0.2					
	goat buttermilk	0.19	35.2	9.8	9.9	24.8	20.3
	goat butter serum	1.01	27.1	11.7	8.2	26.2	26.8
[34]	cream		26.7	7.5	11.7	26.5	20.8
[35]	cream	5.65	17.7	15.4	11.3	33.7	21.8
	butter	5.31	17.7	15.8	11.5	33.3	21.8
	buttermilk	12.4	17	7.1	8.1	46.1	21.7
[12]	buttermilk		8.4	8.2	4.6	51.2	27.6

**Table 4:** SPE conditions applied to purify PLs (the asterisk indicates the procedure that provided the most accurate results).

Reference	Matrix	SPE phase	Solvents for non polar compound elution (*v*/*v*)	Solvents for PL elution (*v*/*v*)
[91]	Egg powder, chicken meat, cheese, salami	Silica	hexane/diethyl-ether (8:2) and (1:1)	methanol and metanol/acetic acid (1% to 5%)
		Aminopropyl (NH_2_)	chloroform/isopropanol (2:1) and diethyl-ether/acetic acid (98:2)	methanol
		Octadecyl (C18)	methanol and methanol/chloroform (4:1)	methanol/water (4:1)
		Octyl (C8) (*)	chloroform/methanol (3:2) and chloroform	methanol
[22]	milk, cream, butter, fresh buttermilk	Silica	hexane/diethyl-ether (8:2) and (1:1)	methanol
		Silica (*)	hexane/diethyl-ether (8:2) and (1:1)	methanol and chloroform/methanol/water (3:5:2)
		Octyl (C8)	chloroform/methanol (3:2) and chloroform	methanol
[90]	cheese	Silica with 20% water	cyclohexane/ethyl acetate (1:1)	ethyl acetate/methanol (1:1), methanol and methanol/water (98:2).
[12]	milk, cream, powdered buttermilk	Aminopropyl (NH_2_-bonded)	chloroform/isopropanol (2:1) and diethyl-ether/acetic acid (98:2)	methanol
		Silica (*)	hexane/diethyl-ether (1:1)	methanol and chloroform/methanol/water (3:5:2)
		Silica	hexane/diethyl-ether (8:2) and (1:1)	methanol and chloroform/methanol/water (3:5:2)
[30]	milk of different species	Silica	hexane/diethyl-ether (8:2 and 1:1)	methanol and chloroform/methanol/water (3:5:2)
[13]	milk of different species	Silica	hexane/diethyl-ether (8:2 and 1:1)	methanol and chloroform/methanol/water (3:5:2)
[31]	milk	Silica	chloroform/methanol (95:5)	methanol and chloroform/methanol/water (3:5:2)

**Table 5:** Analytical conditions applied to quantify and identify PLs (* HILIC = Hydrophilic interaction liquid chromatography).

Reference	Matrix	HPLC Column phase	HPLC mobile phase	ELSD: temperature/pressure or flow	PL identification	Molecules identified in samples
[91]	Egg powder, chicken meat, cheese, salami	Silica	Solvent A: chloroform/metanol/NH_4_OH (80:19.5:0.5) Solvent B: chloroform/methanol/water/NH_4_OH (60:34:5.5:0.5)	60 °C/2 atm	authentic standards	PE, PC, PI, PG, SM, LPC
[22]	milk, cream, butter, fresh buttermilk	Silica	Solvent A: chloroform/metanol/NH_4_OH (80:19.5:0.5) Solvent B: chloroform/methanol/water/NH_4_OH (60:34:5.5:0.5)	50 °C/2.2 bar	authentic standards	PE, PC, PI, PS, SM
[25]	milk, cream, butter, cheese, whey, yoghurt, fermented buttermilk	Silica	chloroform/methanol/buffer (1M formic acid, neutralized to pH 3 with triethylamine) (87.5:12:0.5)	85 °C/1.4 L/min	authentic standards	PE, PC, PI, PS, SM, GluCer, LacCer
[28]	milk of different species, powdered buttermilk	Silica	Solvent A: chloroform/methanol/water (1M formic acid; triethylamine) (87.5:12:0.5). Solvent B: chloroform/methanol/water (1M formic acid; triethylamine) (28:60:12). Solvent C: isooctane/tetrahydrofurane (99:1). Solvent D: 2-Propanol	60 °C/3.5 bar	authentic standards	PE, PC, PI, PS, SM, LacCer
[29]	milk, cheese, butter	Silica	Solvent A: dichloromethane Solvent B: methanol/buffer (7.2 mL acetic acid, 8.0 ml triethylamine and 118 mL HPLC water) (500:21)	65 °C/2.1 L/min	authentic standards	PE, PC, PI, PS, SM, GluCer, LacCer
[30]	milk of different species	HILIC *	Solvent A: acetonitrile Solvent B: acetonitrile/water (2:1)	50 °C/180 KPa	ESI/TOF	PE, PC, PI, PS, SM
[31]	milk	Silica	Solvent A:13% formic acid Solvent B: hexane Solvent C: 2-propanol	CAD detector (see text)	authentic standards	PE, PC, PI, PS, SM

**Table 6:** PL composition, expressed as percent on the total PLs, obtained by 31P NMR analysis.

Reference	Matrix	PC	PE	PI	PS	SM	DHSM	EPLAS	LPC	LPE	PA
[11]	cow milk	26.8	25.8	14.0	1.5	26.8		4.6			0.5
[23]	cow milk	24.0	23.5	12.0	3.6	24.2					
[13]	cow milk	28.7	31.4	3.6	11.2	19.9		4.5			
[34]	cow cream	26.5	26.7	7.5	11.7	20.8	3.9		1.1	1.8	
[11]	ewe milk	23.9	27.5	9.4	3.9	28.3		6.5			0.5
[23]	buffalo milk	21.6	21.8	17.5	5.9	22.1					
[13]	camel milk	19.3	30.0	4.3	9.2	24.6		6.4			0.9
[13]	mare milk	21.3	18.6	6.5	8.1	22.2		3.4	1.0	8.3	3.1
[13]	human milk	24.5	18.3	3.8	8.1	29.7		11.4		2.5	
[34]	buttermilk (lipid fraction)	27.0	25.7	5.8	9.7	20.4	4.6		0.7	1.0	
[34]	buttermilk (direct analysis)	26.4	25.8	7.6	11.5	16.9	4.6		0.7	1.0	

## Abbreviations

DHSM: dihydrosphingomyelin
ELSD: evaporative light scattering detector
ePC: ether phosphatidylcholine
ePE: ether phosphatidylethanolamine
EPLAS: phosphatidylethanolamine plasmalogen
FA: fatty acid
GluCer: glucosylceramide
LacCer: lactosylceramide
LDL: low density lipoproteins: HDL, high density lipoproteins
HPLC: high performance liquid chromatography
LPA: lysophosphatidic acid
LPC: lysophosphatidylcholine
LPE: lysophosphatidylethanolamine
LPS: lysophosphatidylserine
MFGM: milk fat globule membrane
MMPE: monomethylphosphatidylethanolamine
MS: mass spectrometer
NMR: nuclear magnetic resonance
PA: phosphatidic acid
PC: phosphatidylcholine
PE: phosphatidylethanolamine
PE-cer: phosphoethanolamine-ceramide
PG: phosphatidylglycerol
PI: phosphatidylinositol
PL: phospholipid
PLA_2_: phospholipase A_2_
PS: phosphatidylserine
SM: sphingomyelin
